# Effects of a proprietary mixture of extracts from *Sabal serrulata* fruits and *Urtica dioica* roots (WS^®^ 1541) on prostate hyperplasia and inflammation in rats and human cells

**DOI:** 10.3389/fphar.2024.1379456

**Published:** 2024-03-15

**Authors:** Carla Sens-Albert, Sabrina Weisenburger, Beatrix C. König, Silas F. Melcher, Ulrike A. M. Scheyhing, Karin Rollet, Philippe Lluel, Egon Koch, Martin D. Lehner, Martin C. Michel

**Affiliations:** ^1^ Preclinical R&D, Dr. Willmar Schwabe GmbH and Co., KG, Karlsruhe, Germany; ^2^ Urosphere SAS, Parc Technologique Du Canal, Toulouse, France; ^3^ Department of Pharmacology, University Medical Center, Johannes Gutenberg University, Mainz, Germany

**Keywords:** *Sabal serrulata*, *Urtica dioica*, WS^®^ 1541, PRO 160/120, benign prostate hyperplasia, BPH-1 cells, inflammation, epithelial-mesenchymal-transition

## Abstract

**Introduction:** Phytotherapeutics, particularly extracts from *Sabal serrulata* (saw palmetto) fruit or *Urtica dioica* (stinging nettle) root, are popular for the treatment of male lower urinary symptoms in many countries, but their mechanism of action is poorly understood. We performed *in vivo* and *in vitro* studies to obtain deeper insight into the mechanism of action of WS^®^ 1541, a proprietary combination of a *Sabal serrulata* fruit and an *Urtica dioica* root extract (WS^®^ 1473 and WS^®^ 1031, respectively) and its components.

**Methods:** We used the sulpiride model of benign prostatic hyperplasia in rats and tested three doses of WS^®^ 1541 in comparison to finasteride, evaluating weight of prostate and its individual lobes as well as aspects of inflammation, oxidative stress, growth and hyperplasia. In human BPH-1 cells, we studied the effect of WS^®^ 1473, WS^®^ 1031, WS^®^ 1541 and finasteride on apoptosis, cell cycle progression and migrative capacity of the cells.

**Results:** WS^®^ 1541 did not reduce prostate size in sulpiride treated rats but attenuated the sulpiride-induced changes in expression of most analyzed genes and of oxidized proteins and abrogated the epithelial thickening. *In vitro*, WS^®^ 1473 and WS^®^ 1031 showed distinct profiles of favorable effects in BPH-1 cells including anti-oxidative, anti-proliferative and pro-apoptotic effects, as well as inhibiting epithelial-mesenchymal-transition.

**Conclusion:** This data supports a beneficial effect of the clinically used WS^®^ 1541 for the treatment of lower urinary tract symptoms associated with mild to moderate benign prostate syndrome and provides a scientific rationale for the combination of its components WS^®^ 1473 and WS^®^ 1031.

## 1 Introduction

Lower urinary tract symptoms (LUTS) attributed to benign prostatic enlargement (BPE) with bladder outlet obstruction (BOO) due to benign prostatic hyperplasia (BPH) are frequent in men above the age of 40 years, and their prevalence and intensity increase with age (Kupelian et al., 2006). Male LUTS are associated with major bother (Perrin et al., 2005) and impairments of quality of life of patients (Hong et al., 2005; Batista-Miranda et al., 2007) and of their partners (Mitroupoulos et al., 2002). Of note, nocturia is particularly bothersome among the various LUTS (Seki et al., 2008; van Dijk et al., 2010). Various chemically defined treatment options are available for male LUTS and are guideline recommended (Gravas et al., 2022). These include α_1_-adrenoceptor antagonists (e.g., tamsulosin), 5α-reductase inhibitors (e.g., finasteride), phosphodiesterase type 5 (PDE5) inhibitors (e.g., tadalafil); muscarinic receptor antagonists (e.g., tolterodine) or β_3_-adrenoceptor agonists (e.g., mirabegron) can be used to treat storage symptoms that are insufficiently responsiveness to the other drug classes (Bschleipfer et al., 2023; Michel et al., 2023).

While the chemically defined compounds are often effective, many men prefer treatment of their LUTS with various plant extracts (Norton et al., 2018). However, the popularity of phytotherapeutic agents differs considerably between countries ranging between 0% and 40% of men with LUTS across 19 European countries (Cornu et al., 2010). The most frequently used plant extracts are those from *Serenoa repens* (W. Bartram), also known as *Sabal serrulata* (saw palmetto) fruits and *Urtica dioica* (stinging nettle) roots (Fornara et al., 2020; Gravas et al., 2022). Some extracts have been positively evaluated by the European Medicines Agency (European Medicines Agency, 2015). However, the strength of clinical proof differs across preparations.

WS^®^ 1473 is a proprietary ethanolic extract from *Sabal serrulata* fruits and is the active ingredient in the commercially available preparation Prostagutt^®^ uno. WS^®^ 1031 is a proprietary dry extract from *Urtica dioica* roots. WS^®^ 1541, commercially available as Prostagutt^®^ duo, is a proprietary combination of WS^®^ 1473 and WS^®^1031 (see section 2.1). The efficacy and safety of WS^®^ 1541 has been demonstrated in randomized, double-blind controlled trials comparing it to placebo (Metzker et al., 1996; Lopatkin et al., 2005), to finasteride (Sökeland and Albrecht, 1997), and to tamsulosin (Engelmann et al., 2006). One of the placebo-controlled trials has also reported on 96-month follow-up data that confirmed the findings from the prior 24-week observation period (Lopatkin et al., 2007). A pooled analysis of the four controlled trials has reported a 69% responder rate to PRO 160/120 (WS^®^ 1541) improving nocturnal voiding frequency (Oelke et al., 2014).

Various potential mechanisms have been proposed to explain the clinically observed efficacy of *Sabal serrulata* and *Urtica dioica* extracts (Koch, 2001; Buck, 2004; Pigat et al., 2019; Csikós et al., 2021), but clear proof has remained elusive. Therefore, the present experiments were designed to obtain deeper insight into the mechanisms of action of WS^®^ 1541 and the relative contributions of WS^®^ 1473 and WS^®^ 1031. For this purpose, we have performed an exploratory study on the treatment effect of different doses of WS^®^ 1541 in an *in vivo* rat model of sulpiride-induced BPH (Van Coppenolle et al., 2000; Van Coppenolle et al., 2001) followed by an evaluation of effects on parameters of epithelial-mesenchymal transition, apoptosis, proliferation, and oxidative stress of individual extracts WS^®^ 1473 and WS^®^ 1031 and the combination WS^®^ 1541 in an *in vitro* model of BPH using human BPH-1 cells (Hayward et al., 1995; Wu et al., 2007; Yang et al., 2017).

## 2 Materials and methods

### 2.1 Extracts and chemicals

WS^®^ 1541 contained in PRO 160/120 is a proprietary combination of 160 mg extract from saw palmetto fruits (Serenoa repens (Bartram) J.K.Small, syn. *Sabal serrulata* (Michx.) Schult.f), (DER 10–14.3:1), extraction solvent: ethanol 90% (m/m) (WS^®^ 1473) and 120 mg dry extract from stinging nettle root (*Urtica dioica* L.), (DER 7.6–12.5:1), extraction solvent: ethanol 60% (m/m) (WS^®^ 1031). The plant material was fully authenticated by morphologic and analytical means complying to the European pharmacopoeia (Ph. Eur. 11.0) by the internal pharmacognosy and quality control departments of Dr. Willmar Schwabe GmbH and Co. KG prior to extraction and specimens are stored for 10 years. Individual batches of the two extracts were taken from regular GMP production batches, with Urtica extract (batch W814698) containing 82.5 ppm scopoletin and Sabal extract (batch EXCh.952) containing 82.0% fatty acids and 0.3% sterols calculated as β-sitosterol as specified in the certificates of analyses. Furthermore, the extracts were characterized by three chemical fingerprinting methods (Supplementary Figure S1), with high performance thin layer chromatography (HPTLC) and nuclear magnetic resonance spectroscopy (NMR) used for both extracts, while gas chromatography flame ionization detector (GC-FID) was exclusively used for Sabal extract and liquid chromatography high resolution mass spectrometry (LC-HRMS) solely used for Urtica extract due to the respective phytochemical nature of the two extracts. The aforementioned characterization of the plant material, extraction process and final extract comply in most instances with the requirements of the Consensus statement on the Phytochemical Characterization of Medicinal Plant extracts (Heinrich et al., 2022).

Sulpiride was obtained from Sigma. Finasteride (Think Chemical Co., Ltd.) served as an active control in some experiments.

### 2.2 *In vivo* study in rats

#### 2.2.1 Animals

The experimental protocols had been approved by the CEEA-122 ethical committee for protection of animals used for scientific purposes (n° CEEA-122 2014–34) and were carried out in accordance with the European Community Council Directive 2010/63/UE. They were performed in 2015 with a valid license for experiments on vertebrate animals, issued by the French Ministry for Agriculture and Fisheries (Urosphere, N°31–1155-46).

Adult male Wistar rats (Crl:WI(Han)) 8–9 weeks of age at the beginning of the experiments were obtained from Charles River Laboratories, L´Arbresle, France. They were housed in groups of two to three in polysulfone type Sealsafe GR900 cages (Tecniplast) on beds of wood chips (Toplit-Select Fine, Safe) with free access to food (Rodent Maintenance Diet A04/10 from Safe) and water (0.2 µm filtered water) and acclimatized to the laboratory conditions for at least 3 days before the start of any experiments. All animals were supplied with appropriate environmental enrichment (Play Tunnel, Plexx) in accordance with the European Community Council Directive 2010/63/UE. The animal house was maintained under artificial lighting (12 h) between 7:00 a.m. and 7:00 p.m. at a controlled ambient temperature of 22°C ± 2°C and a relative humidity of 55% ± 10%.

**FIGURE 1:**
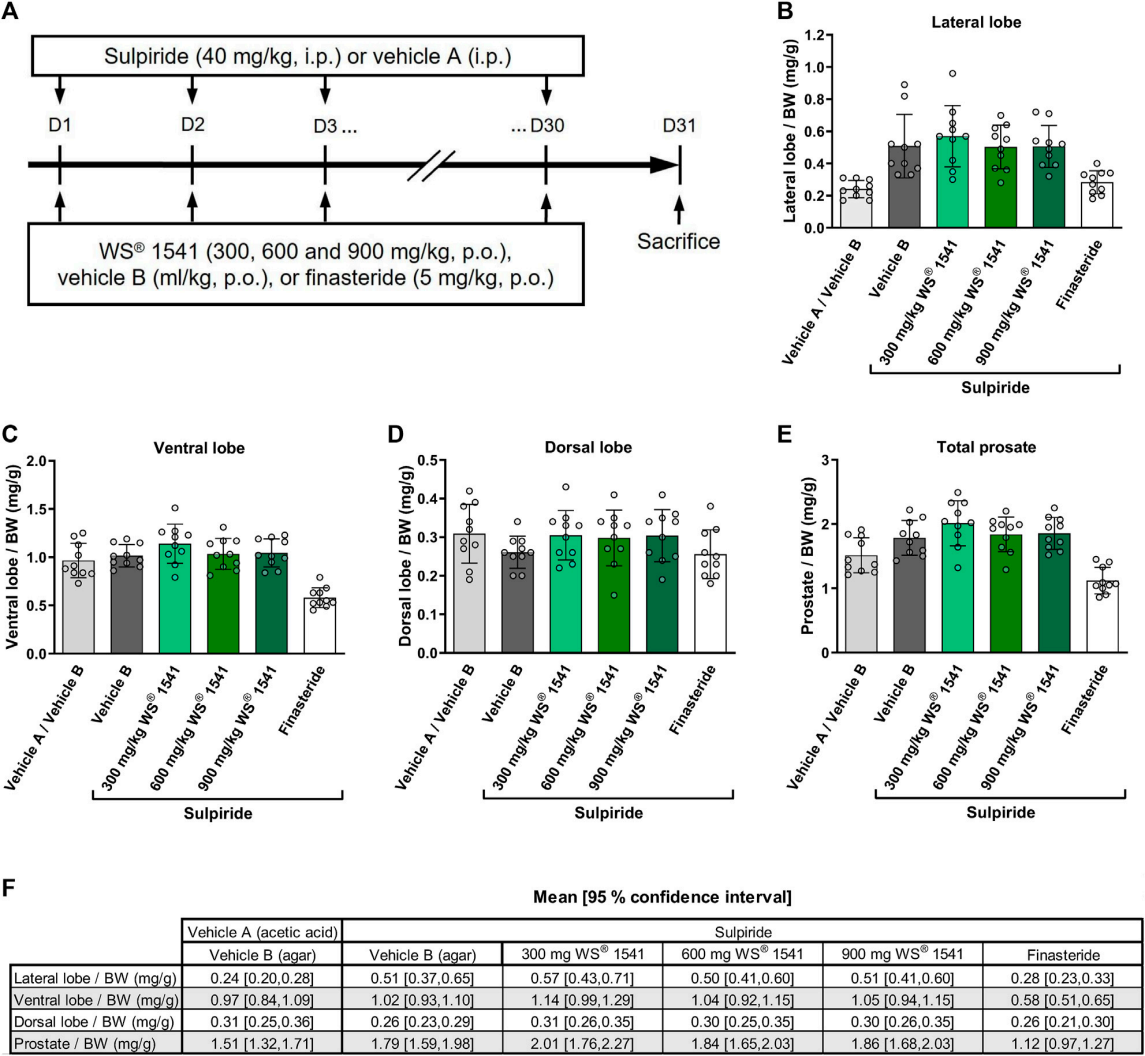
**(A)** Experimental outline of the sulpiride-induced prostate hyperplasia model in rats. Sulpiride (40 mg/kg) or vehicle A (acetic acid 1%) was administered intraperitoneally (i.p.) once a day for 30 consecutive days. The proprietary combination of *Sabal serrulata* fruit and *Urtica dioica* root extract WS^®^ 1541 (300, 600 and 900 mg/kg), finasteride (5 mg/kg) or vehicle B (0.2% agar suspension with 1% Tween 80 in distilled water) was given orally daily for 30 consecutive days. Weight normalized to total body weight (BW) of **(B)** lateral, **(C)** ventral, and **(D)** dorsal prostate lobes showing specific induction of hyperplasia in the lateral lobe rather than in the ventral and dorsal lobe (n = 10). **(E)** Total prostate weight normalized to BW of different treatment groups (n = 10). **(F)** Tabular presentation of 95% confidence intervals for the data shown in Figures 1B–E. BW was similar in all groups (Online Supplementary Figure S1). No *p*-values were calculated as these measurements served as model validation only.

Sulpiride (40 mg/kg) or vehicle A (acetic acid 1%) was administered intraperitoneally (i.p.) at a volume of 1 ml/kg once a day for 30 consecutive days (a.m.). WS^®^ 1541 (300, 600 and 900 mg/kg), finasteride (5 mg/kg) or vehicle B (0.2% agar suspension with 1% Tween 80 in distilled water) was orally (p.o.) administered once a day (a.m.) at a volume of 5 ml/kg for 30 consecutive days (Figure 1A). At the end of treatment series, rats were sedated by an injection of sodium pentobarbital at 54.7 mg/ml (0.5 ml/rat) and sacrificed by cervical dislocation. Each prostate lobe was dissected and weighed. Left and right lateral lobes were maintained on dry ice during measurement and stored at—80°C for later use.

#### 2.2.2 RNA isolation, cDNA synthesis, and quantitative real-time PCR

RNA was isolated from snap frozen tissue from left lateral lobes using SV Total RNA Isolation System (Z3105, Promega) and cDNA was transcribed from 0.5 µg RNA with iScript Reverse Transcription Supermix for RT-qPCR (170–8841, Bio-Rad), both according to manufacturer’s instructions. For SYBR Green based quantitative real-time PCR Sso Advanced Universal SYBR Green Supermix was applied (172–5271; Bio-Rad).

Expression of mRNA in rat prostate tissue was evaluated using Bio-Rad pretested primers and amplification was normalized to the reference gene *Gapdh* (qRnoCID0057018). Primers for rat samples were as follows: *Ccl2* (qRnoCED0009272), *Ccl7* (qRnoCED0005636), *Cox2* (qRnoCED0052820), *Egf* (qRnoCED0004669), *Egr1* (qRnoCED0001041), *Fgf2* (qRnoCID0003540), *Icam1* (qRnoCED0005284), *Ikbkb* (qRnoCID00051680), *Il1b* (qRnoCID0004680), *Il2* (qRnoCED0006493), *Il4* (qRnoCID0002254), *Il6* (qRnoCID0053166), *Il7* (qRnoCID00062112), *Il13* (qRnoCID0008414), *Il15* (qRnoCID0009113), *Il16* (qRnoCID0005696), *Il21* (qRnoCID0008854), *Ifng* (qRnoCID0006836), *Nos2* (qRnoCID0017722), *Tgfb* (qRnoCID0009191), *Tnf* (qRnoCED0009117), *Vegfa* (qRnoCED0002159).

#### 2.2.3 H&E staining

Cryosections (5 µm) from left lateral prostate lobes were fixed with 4% paraformaldehyde (PFA) (Roth, 0335.1) for 5 min. PFA was removed and sections were washed with 1x phosphate buffered saline (PBS). Nuclei were stained using gill hematoxylin (VWR, 105174) for 2 s followed by brief washing in tap water and 10 s in 1% acetic acid. After blueing in tap water, sections were washed in distilled water and subsequently incubated for each 60 s in 0.5% eosin G-solution (VWR, 102439), 1% eosin G-solution (VWR, 117081) and eosin-phloxine solution (VWR, 102480). Sections were dehydrated in a serial dilution (70%/96%/100% ethanol), cleared using Neo-Clear (VWR, 109843) for 15 s and mounted in Eukitt Quick hardening mounting medium (Sigma Aldrich, 03989).

#### 2.2.4 OxyIHC staining

The Millipore OxyIHC Oxidative Stress Detection Kit (S7450, Merck) was used to stain 5 µm cryosections from left lateral prostate lobes according to the manufacturer’s instructions except for antigen retrieval. Antigen retrieval was accomplished by microwaving the sections in sodium citrate buffer (10 mM Sodium citrate, 0.05% Tween 20, pH 7.0) for 15 min. For each prostate sample three sections on a slide were processed, one for specific staining, one for secondary antibody control and another one as derivatization control. Following the staining procedure, sections were mounted in Neo-Clear (109843, VWR). Images were taken at ×10 magnification on Zeiss Axio Observer with an Axiocam 105 color. Stained area was quantified using the 8-bit threshold tool of ImageJ (Wayne Rasband, National Institutes of Health, Bethesda, MD, United States of America).

### 2.3 *In vitro* studies in BPH-1 cells

#### 2.3.1 Source and culturing of BPH-1 cells

BPH-1 cells were purchased from DMSZ (Cat# ACC-143, RRID: CVCL_1091) in 2005 and were cultured in RPMI-1640 (R8758, Sigma) containing 10% FBS (F4135, Sigma) and 1% antibiotic antimycotic solution (A5955, Sigma). Cells were regularly negative tested for *mycoplasma* contamination using MycoStrip™ - *Mycoplasma* Detection Kit (MycoStrip™ 100, InvivoGen).

#### 2.3.2 Stimulation of BPH-1 cells

To assess changes in mRNA expression of *TGFB1*, *TGFB2*, *TGFB3*, *TGFBR1*, and *TGFBR2*, 200,000 BPH-1 cells per well were seeded in a 24-well plate in the above-mentioned media. After adherence overnight, cells were washed and stimulated with either a serial dilution of Urtica (WS^®^ 1031) or Sabal (WS^®^ 1473) (0.3 μg/mL, 1 μg/mL, 3 μg/mL, 10 μg/mL, 30 μg/mL, 100 μg/mL) for 24 h followed by cell harvest for RNA isolation and procession for qPCR analysis. Briefly, cells were washed once with ice-cold PBS and directly lysed in RNA lysis buffer supplied in the RNA isolation kit used (see section 3.3.7).

#### 2.3.3 ROS assay

25.000 BPH-1 cells per well were seeded in Corning^®^ 96-well Flat Clear Bottom Black Polystyrene TC-treated Microplates (3603, Corning) and cultured for 24 h. Medium was removed and cells were loaded with 25 µM DCFDH/H2DCFDA for 45 min followed by treatment with either a serial dilution of Urtica (WS^®^ 1031) or Sabal (WS^®^ 1473) (0.3 μg/mL, 1 μg/mL, 3 μg/mL, 10 μg/mL, 30 μg/mL, 100 μg/mL) or 25 μg/mL Sabal, 21.9 μg/mL Urtica (according to their presence in WS^®^ 1541), 25 μg/mL WS^®^ 1473 + 21.0 μg/mL WS^®^ 1031 or 100 µM finasteride were added. DMSO was used as solvent and added accordingly at 0.25% as control treatment. Dilutions for treatment were prepared in absence of Phenol red in Dulbecco’s Modified Eagle’s Medium/Nutrient Mixture F-12 Ham (D-6434, Sigma-Aldrich). DCF-production was measured 1 h after adding the treatment at 535 nm using a Versamax Microplate Reader (Molecular Devices).

#### 2.3.4 Annexin V apoptosis assay

For assaying apoptosis, 100.000 BPH-1 cells per well were seeded in a 6-well format and cultured overnight. Cells were treated for 2 h with DMSO 0.25%, WS^®^ 1473 25 μg/mL, WS^®^ 1031 21.9 μg/mL according to their presence in WS^®^ 1541, 25 μg/mL WS^®^ 1473 + 21.0 μg/mL WS^®^ 1031 or 100 µM finasteride and detached with Accutase Cell Detachment Solution, w: 0.5 mM EDTA, w: Phenol red (P10-21500, PAN-Biotech). Following, cells were stained for signs of apoptosis using the APC Annexin V Apoptosis Detection Kit with PI (640932, BioLegend) according to manufacturer’s instructions. FACS-analysis was performed at a NovoCyte flow cytometer and evaluated using the NovoExpress software.

#### 2.3.5 5-Ethynyl-2′-dioxyuridine (EdU) proliferation assay

BPH-1 cells were seeded (100.000 cells/well, 6 well plate) and cultured overnight. Treatment occurred for 48 h with DMSO 0.25%, WS^®^ 1473 25 μg/mL, WS^®^ 1031 21.9 μg/mL according to their presence in WS^®^ 1541, 25 μg/mL WS^®^ 1473 + 21.0 μg/mL WS^®^ 1031 or 100 µM finasteride. Cell staining was performed using the EdU Flow Cytometry Kit 488 (BCK-FC-488–50, Sigma). Briefly, EdU was added for 1 h before harvesting the cells and followed by the EdU-detection procedure and 7AAD staining. Analysis was performed at a Novocyte flow cytometer using the NovoExpress software.

#### 2.3.6 Migration assay

BPH-1 cells were seeded in a 2 well silicone insert with a defined 500 μm cell-free gap (81176, Ibid) each 50.000 cells/well and allowed to adhere overnight. Inlays were removed with sterile forceps and images were taken directly afterwards (t = 0). Either a serial dilution of Urtica (WS^®^ 1031) or Sabal (WS^®^ 1473) (0.3 μg/mL, 1 μg/mL, 3 μg/mL, 10 μg/mL, 30 μg/mL, 100 μg/mL) or 25 μg/mL Sabal, 21.9 μg/mL Urtica according to their presence in WS^®^ 1541 were added. DMSO was used as solvent for WS^®^ 1031 and WS^®^ 1473 and added accordingly at 0.25% as control treatment. Cells were cultured for another 24 h followed by image capture (t = 24 h). Images were taken in ×5 magnification at a Primovert KMAT microscope using Axiocam 208 color (Zeiss). Cell-free area was measured using ImageJ for t = 0 and t = 24 h and percentage of initially free area was calculated and is presented in the figures.

#### 2.3.7 RNA isolation, cDNA synthesis and mRNA analysis

RNA from treated BPH-1 cells was extracted using the GenUP™ Total RNA Kit (BRO700903, Biozym Scientific GmbH) and cDNA was transcribed from 0.5 µg RNA with iScript Reverse Transcription Supermix for RT-qPCR (170–8841, Bio-Rad), both according to manufacturer’s instructions. For SYBR Green based quantitative real time PCR Sso Advanced Universal SYBR Green Supermix was applied (172–5271; Bio-Rad).

For qPCR analysis in human BPH-1 cells the following primers were used (Chen et al., 2015): human *TGFB1*_fwd 5′-tcg​cca​gag​tgg​tta​tct​t-3′, *TGFB1*_rev 5′-tag​tga​acc​cgt​tga​tgt​cc-3′

Human *TGFB2*_fwd 5′-aca​ctc​agc​aca​gca​ggg​t-3′, *TGFB2*_rev 5′-ttg​gga​cac​gca​gca​ag-3′

Human *TGFB3*_fwd 5′-tga​gtg​gct​gtt​gag​aag​aga-3′, *TGFB3*_rev 5′-att​gtc​cac​gcc​ttt​gaa​tt-3′

Human *TGFBR1*_fwd 5′-cag​agc​tgt​gaa​gcc​ttg​a-3′, *TGFBR1*_rev 5′-tgc​ctt​cct​gtt​gac​tga​gt-3′

Human *TGFBR2*_fwd 5′-atg​aca​tct​cgc​tgt​aat​gc-3′, *TGFBR2*_rev 5′-gat​gcc​ctg​gtg​gtt​ga-3´.

Expression was normalized to human *HPRT* qHsaCID0016375 (Bio-Rad). Assays were either run on a thermal Cycler CFX Connect or on a CFX Opus 96 (Bio-Rad) with the following cycle protocol: 2 min 95°C, 40x (5 s 95°C, 30 s 60°C); melt curve: 65°C–95°C; 0.5°C increments, 6 s per step.

### 2.4 Data analysis and presentation

Data were analyzed using GraphPad Prism 10 (GraphPad Software, La Jolla, CA) and are presented as mean values ±standard deviation (SD). Experimental groups were compared using one-way analysis of variance (ANOVA) followed by Dunnett post-tests. In the *in vivo* study, vehicle A/vehicle B and each active treatment group were compared to the sulpiride/vehicle B group. In the *in vitro* study, untreated cells and those with active treatments were compared to the DMSO group unless stated otherwise. In line with the exploratory character of the experiments and recent guidelines (Michel et al., 2020a), calculated *p*-values should not be interpreted as hypothesis testing and only as descriptive. Relevant *p*-values <0.05 are presented in the figures.

## 3 Results

### 3.1 *In vivo* rat study

Neither sulpiride nor any of the WS^®^ 1541 doses, nor finasteride affected body weight (Supplementary Figure S2). As reported by others (Van Coppenolle et al., 2000; Van Coppenolle et al., 2001), treatment with sulpiride for 30 days approximately doubled the weight of the lateral prostate lobes while not affecting that of the ventral and dorsal lobe or total prostate/body weight ratio (Figures 1B–E). Treatment with WS^®^ 1541 did not affect the weight of the lateral or any other lobe relative to vehicle B. In contrast, the active control finasteride reduced the weight of the lateral and ventral lobe and total prostate weight. Thus, our model recapitulates the known phenotype of the sulpiride model and the established effects of the active control finasteride.

**FIGURE 2:**
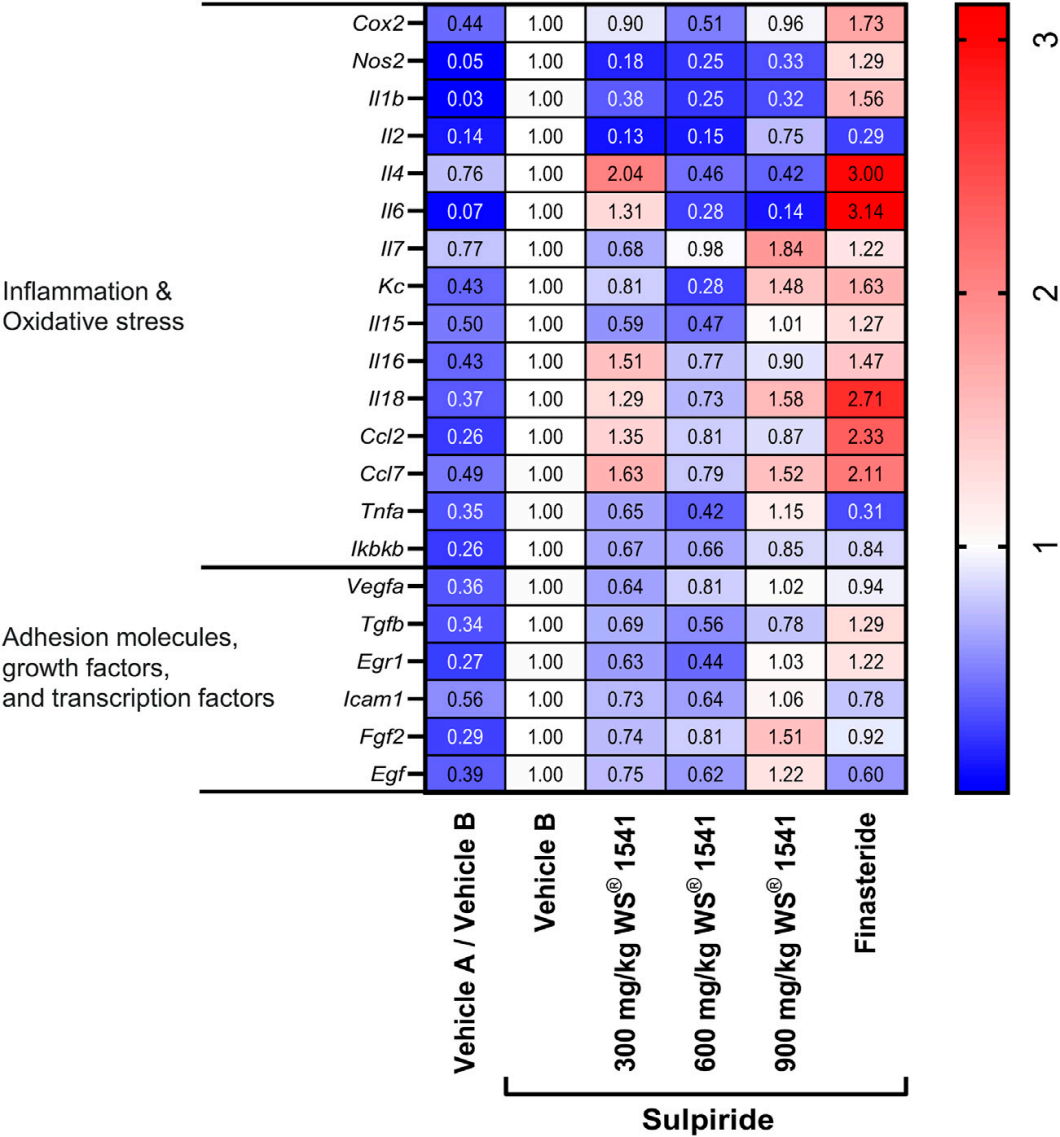
Heatmap on mRNA expression of indicated genes in lateral lobes of the prostate from treated rats normalized to the reference gene *Gapdh* (n = 8–10). The expression level of each gene was set to one in the vehicle B treated sulpiride group. Changes in expression vs. the sulpiride-treated vehicle B group are shown in the individual fields.

We then used the validated model to explore effects of the various treatments on the expression of various genes related to oxidative stress and/or inflammation (*Cox2*, *Nos2*, *Il1b*, *Il2*, *Il4*, *Il6*, *Il7*, *Kc*, *Il15*, *Il16*, *Il18*, *Ccl2*, *Ccl7*, *Tnfa*, and *Ikbkb*), adhesion molecules, growth factors and transcription factors (*Vegfa*, *Tgfb*, *Egr1*, *Icam1*, *Fgf2* and *Egf*) in lateral lobe tissue. The expression of the reference gene *Gapdh* was similar in all groups (Supplementary Figure S3), and all target gene expressions were presented relative to that of *Gapdh*. Relative to the sulpiride/vehicle B group, all target genes were expressed less in the vehicle A/vehicle B group (Figure 2), implying that induction of a lateral lobe enlargement was associated with an upregulation of all analyzed target genes. Treatment with WS^®^ 1541 tended to attenuate the sulpiride-induced gene expression in the majority of analyzed genes, indicative of an overall normalization of expression with a profile more similar to control animals. There was no clear evidence of a dose-response curve. However, treatment with finasteride appeared to exacerbate parameters indicative of inflammation and oxidative stress. Except for *Il2* and *Tnfa*, finasteride increased the pro-inflammatory expression profile as seen by an upregulation of *Cox2*, *Il1b*, *Il4*, *Il6*, *Kc*, *Il18*, *Ccl2* and *Ccl7*. Focusing on adhesion molecules, transcription and growth factors, treatment with WS^®^ 1541 attenuated increased expression levels of *Vegfa*, *Tgfb*, *Egr1*, *Icam1*, *Fgf2* and *Egf* seen in sulpiride treated control animals. Finasteride treatment showed a similar expression pattern to the sulpiride/vehicle B group but elevated levels of *Tgfb* and *Egr1* expression.

**FIGURE 3:**
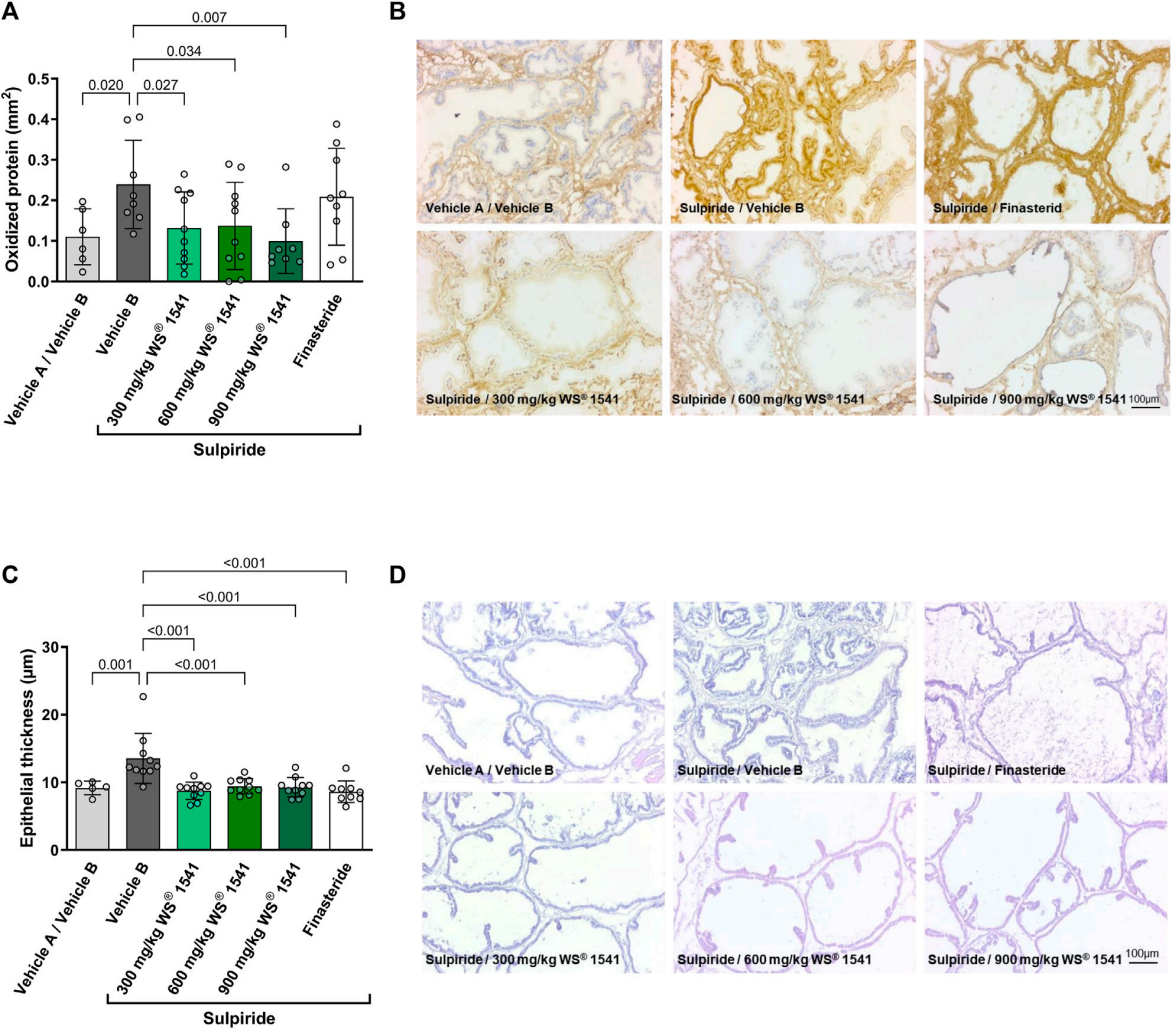
**(A)** Quantification of immune histological staining (OxyIHC) for oxidized proteins (n = 6/8/10/10/8/9). **(B)** Representative images showing brownish staining for oxidized proteins and nuclei in blue (scale 100 µm). **(C)** Histological evaluation of epithelial thickness in H&E stained sections from lateral lobes of treated rats (n = 5/10/10/10/10/9). **(D)** Representative images of H&E stained cryosections of left lateral lobes (scale 100 µm).

Sulpiride-treated rats exhibited an approximate doubling of oxidized protein as assessed by immunohistochemistry (Figures 3A, B). While this parameter was not affected by finasteride, all three doses of WS^®^ 1541 inhibited the increase in oxidized protein to levels similar to the double control animals.

Treatment with sulpiride induced epithelial hypertrophy as evidenced by an increase in epithelial thickness by approximately 50%. This increase was completely prevented by all treatments (Figures 3C, D).

### 3.2 *In vitro* experiments in human BPH-1 cells

**FIGURE 4:**
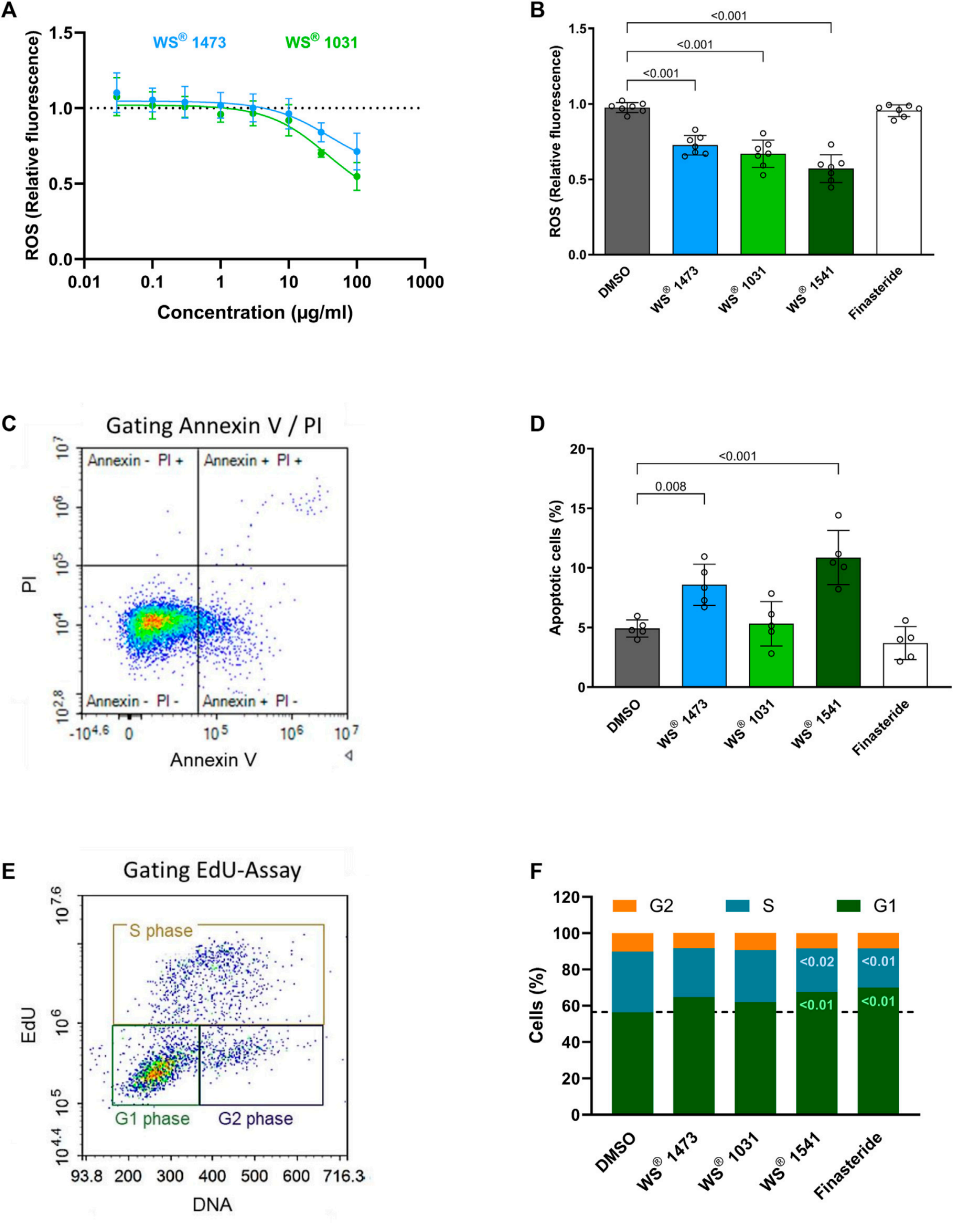
**(A)** Dose-response curve of Sabal (WS^®^ 1473) and Urtica (WS^®^ 1031) extracts on reactive oxygen species (ROS) production in treated BPH-1 cells measured based on H2-DCF-DA to fluorescent DCF conversion (n = 4 independent experiments). **(B)** Quantification of ROS in BPH-1 cells at definite concentrations (25 μg/mL WS^®^ 1541, 21.9 μg/mL WS^®^ 1031) according to their presence in WS^®^ 1541 (25 μg/mL WS^®^ 1541 + 21.9 μg/mL WS^®^ 1031) (n = 7 independent experiments). **(C)** Exemplary gating of BPH-1 cells stained for Annexin V and with propidium iodide (PI). **(D)** Quantification of the sum of Annexin V+ and Annexin V+/PI + BPH-1 cells after 2 h of treatment with DMSO, Finasteride, Sabal (WS^®^ 1473), Urtica (WS^®^ 1031) or its combination (WS^®^ 1541) (n = 5 individual experiments). **(E)** Gating strategy on EdU and DNA staining of BPH-1 cells to differentiate between G_1_-, S- and G_2_-phase of the cell cycle. **(F)** Percentage of BPH-1 cells in G_1_-, S- or G_2_-phase during cell cycle progression after treatment with DMSO, finasteride, Sabal (WS^®^ 1473), Urtica (WS^®^ 1031) or the combination of Sabal + Urtica (WS^®^ 1541) (n = 5 individual experiments).

For further evaluation of potential mechanisms of action underlying the observed treatment effect of WS^®^ 1541 against oxidative, inflammatory and hyperproliferative changes, the combination product WS^®^ 1541 and its individual extract components WS^®^ 1473 and WS^®^ 1031, were tested in a set of *in vitro* experiments using BPH-1 cells. In this immortalized luminal epithelial cell line isolated from a 68-year-old patient with benign prostrate hyperplasia, both single extracts concentration-dependently reduced basal production of reactive oxygen species (Figure 4A). To reflect the relative abundance of the individual extracts in the clinically used combination product, a separate experiment compared 25 μg/mL WS^®^ 1473, 21.9 μg/mL WS^®^ 1031 and 46.9 μg/mL WS^®^ 1541 as well as 100 µM finasteride (Figure 4B). This experiment confirmed the lack of effect of finasteride on ROS production. In contrast, both WS^®^ 1473 and WS^®^ 1031 reduced ROS abundance and the extract combination WS^®^ 1541 caused a more prominent inhibition than either extract alone.

We next examined the effects of the extracts and of finasteride on apoptosis and cell cycle progression. While neither finasteride nor WS^®^ 1031 affected the amount of annexin V^+^ cells, WS^®^ 1473 and WS^®^ 1541 stimulated apoptosis (Figures 4C, D). Using the EdU assay to assess possible cell cycle effects, we found that treatment with finasteride resulted in increased cells numbers in G_1_-phase. The single extracts WS^®^ 1031 and WS^®^ 1473 showed little effect on cell cycle progression. However, their combination in WS^®^ 1541 increased the amount of cells in G_1_-phase as seen with finasteride, indicating possible synergism of both extracts. Consistent with these results, finasteride and WS^®^ 1541 decreased the cell number in S-phase, thus inhibiting cell cycle progression by holding the cells in G_1_-phase. No change was observed for any of the treatments on the G_2_ phase (Figures 4E,F).

**FIGURE 5:**
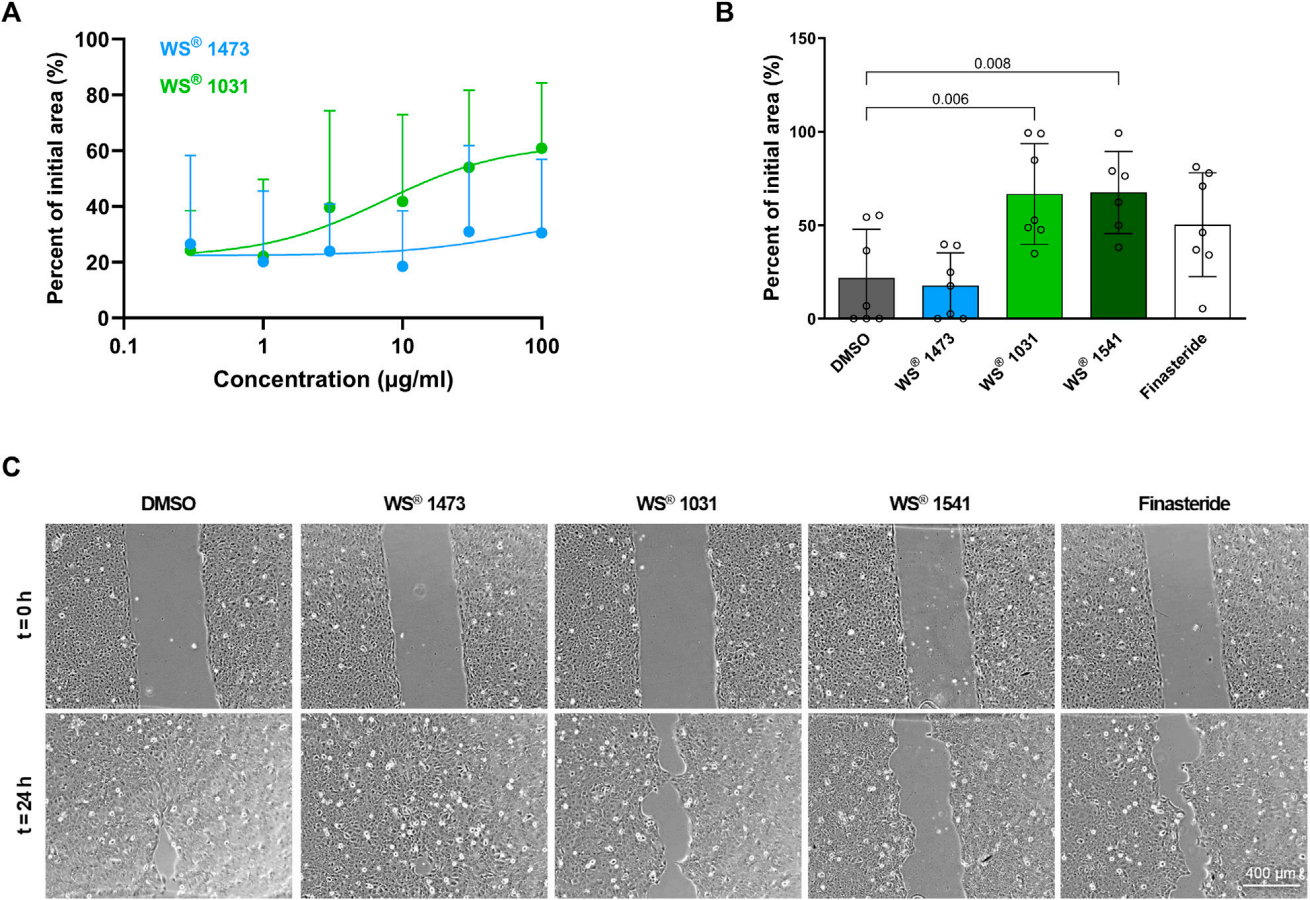
**(A)** Concentration-dependency of Sabal (WS^®^ 1473) and Urtica (WS^®^ 1031) extracts on migration of BPH-1 cells (n = 9 independent experiments). **(B)** Quantification of the migration assay performed with BPH-1 cells in the presence of DMSO, finasteride, 25 μg/mL Sabal (WS^®^ 1473) or 21.9 μg/mL Urtica (WS^®^ 1031) or its combination (WS^®^ 1541; 25 μg/mL WS^®^ 1473 and 21.9 μg/mL WS^®^ 1031) showing inhibition of migration in response to Urtica (WS^®^ 1031) or Sabal + Urtica (WS^®^ 1541) (n = 7 individual experiments). **(C)** Representative images of the migration assay.

To obtain insight into the effect of the treatments on cell migration as a marker for epithelial-mesenchymal-transition (EMT), a gap-closing assay was performed. Concentration-response experiments showed that WS^®^ 1473 was ineffective whereas WS^®^ 1031 concentration-dependently inhibited migration (Figure 5A). In another experimental setting with constant concentrations, we confirmed that WS^®^ 1473 was ineffective in this assay, while WS^®^ 1031, WS^®^ 1541 and finasteride decreased migration retaining more of the initial cell free area (Figures 5B, C).

**FIGURE 6:**
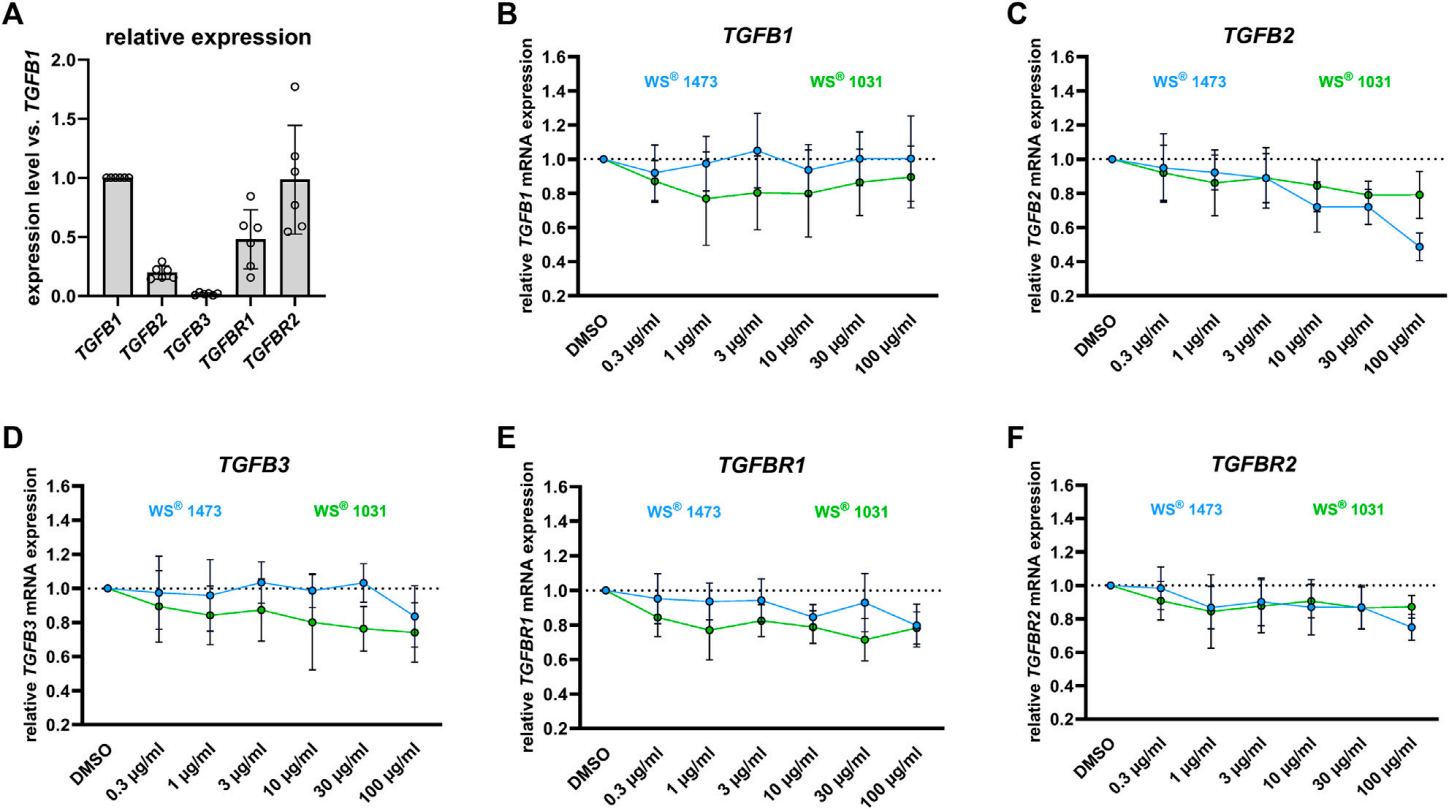
**(A)** Relative expression levels of *TGFb2*, *TGFb3* and the TGF-β receptors *TGFbR1* and *TGFbR2* in BPH-1 cells treated for 24 h relative to that of *TGFb1* (n = 6 independent experiments). Impact of Sabal (WS^®^ 1473) and Urtica (WS^®^ 1031) extracts on the mRNA expression of **(B)** *TGFb1*, **(C)** *TGFb2*, **(D)** *TGFb3* and the TGF-β receptors **(E)** *TGFbR1* and **(F)** *TGFbR2* in BPH-1 cells treated for 24 h (n = 6 independent experiments).

As TGF-β is a key driver of EMT, we examined the effect of WS^®^ 1473 and WS^®^ 1031 on mRNA expression of the three TGF-β isoforms and its two receptors. We observed no changes due to different treatments in mRNA expression of *HPRT* verifying *HPRT* as suitable reference gene (Supplementary Figure S4). All three TGF-β isoforms and their receptors were expressed in BPH-1 cells. To compare expression levels of the different isoforms, we normalized all expression levels to *TGFB1* expression, which showed the highest expression level. Expression levels of the other TGF-β isoforms normalized to *TGFB1* were much lower with 0.20 and 0.02 for isoforms 2 and 3, respectively. Concerning TGF-β receptors, a 2-fold higher expression level was found for *TGFBR2* vs. *TGFBR1* with normalized expression levels of 0.99 and 0.48, respectively (Figure 6A). WS^®^ 1031 moderately decreased expression of *TGFB1*, *TGFB2*, *TGFB3* and *TGFBR1* without a clear concentration-response effect. WS^®^ 1473 had no effect on expression of either *TGFB1* or *TGFB3* at concentrations up to 30 μg/mL, but concentration-dependently reduced the mRNA expression of *TGFB2*. Both WS^®^ 1031 and WS^®^ 1473 had a minor effect on the expression of *TGFBR2* (Figures 6B–F). F-Actin-Polymerization was not affected by WS^®^ 1473 and WS^®^ 1031 treatment (Supplementary Figure S5).

## 4 Discussion

WS^®^ 1541, containing a fixed combination of proprietary extracts from *Sabal serrulata* fruits and *Urtica dioica* roots, is the active pharmaceutical ingredient in herbal medicinal products approved for the treatment of lower urinary tract symptoms attributed to BPH. A number of preclinical studies have been conducted with either *Sabal serrulata* or *Urtica dioica* extract but much less is known concerning the pharmacological activity of the combination and the assessment of the relative contributions of the individual extracts.

Our experiments were designed to obtain deeper insight into the mechanisms of action of WS^®^ 1541 and the relative contributions of WS^®^ 1473 and WS^®^ 1031. For this purpose, we have performed two exploratory studies, one on WS^®^ 1541 in an *in vivo* rat model of sulpiride-induced BPH (Van Coppenolle et al., 2000; Van Coppenolle et al., 2001) and one comparing WS^®^ 1541, WS^®^ 1473 and WS^®^ 1031 in an *in vitro* model of BPH, human BPH-1 cells (Hayward et al., 1995; Wu et al., 2007; Yang et al., 2017).

### 4.1 Limitation of methods

Treatment with the dopamine D_2_ receptor antagonist sulpiride leads to hyperprolactinemia. While this is an adverse effect in clinical use, it can be leveraged to induce BPH in rats (Van Coppenolle et al., 2000; Van Coppenolle et al., 2001). The design of the present *in vivo* study in rats follows that of a previous similar study in mice in which hyperprolactinemia was induced by transgenic expression of prolactin (Pigat et al., 2019). Of note, the sulpiride model induces an enlargement of the lateral lobe whereas the ventral and dorsal lobes are insensitive to sulpiride-induced hyperprolactinemia (Van Coppenolle et al., 2000; Van Coppenolle et al., 2001). This phenotype with preferential enlargement of the lateral lobes was recapitulated in the present study, thereby validating the sulpiride treatment.

Our *in vivo* study had a preventive design (i.e., treatment starting concomitant with sulpiride), which may be more sensitive to treatment effects than designs in which medication was administered after prostatic enlargement had manifested.

Several measures were implemented to increase data robustness of our experiments. First, we used finasteride as a positive control in most of our experiments in high doses or concentrations (5 mg/kg/day or 100 µM) to exclude false negative effects due to underdosing. The 5α-reductase inhibitor finasteride is an established and guideline-recommended medical treatment to delay progression of BPH (Gravas et al., 2022). Second, we applied three extract doses in the *in vivo* study and several extract concentrations in the *in vitro* experiments to obtain more robust data. As high concentrations or doses of plant extracts can have non-specific effects due to the presence of toxoflavins, enones, quinones or catechols (Baell and Walters, 2014), it is important to demonstrate that the observed effects have specificity and are not due to non-specific effects. Therefore, we specifically compared how the single extracts WS^®^ 1473 and WS^®^ 1031 contribute to the effects of the clinically used WS^®^ 1541 *in vitro*. Third, while it is usually assumed that reference gene expression used for normalization of mRNA data is stable, examples of regulation have been reported for all commonly used reference genes (Hendriks-Balk et al., 2007; Bollmann et al., 2012; Michel-Reher and Michel, 2015). Therefore, we explicitly tested the reference gene used for normalization purposes in our studies; *Gapdh* was stably expressed within the *in vivo* experimental setting and *in vitro HPRT* expression in BPH-1 cells was irrespective of treatment. Fourth, based on various guidelines on data robustness (Vollert et al., 2020; Vollert et al., 2022), we classify our experiments as exploratory, i.e., not designed to test a specific statistical null hypothesis. Rather, we applied multiple complementary approaches to reach robust conclusion. Finally, in line with the exploratory character of our experiments and with recommendation from leading statisticians (Amrhein et al., 2019; Michel et al., 2020b), we did not perform hypothesis-testing statistical analysis. Rather, all calculated *p*-values should be interpreted as descriptive only.

These aspects should be considered in the interpretation of our data. In the following we will discuss our findings based on mechanistic pathways by integrating those from the *in vivo* and *in vitro* studies, followed by a discussion of the individual contributions of WS^®^ 1473 and WS^®^ 1031 to the overall effects of the clinically used WS^®^ 1541.

### 4.2 Effects on prostate growth

As male LUTS are assumed to be caused by bladder outlet obstruction due to benign prostatic enlargement, reducing prostate growth or even shrinking prostate size is a mechanism of action useful for delaying BPH progression and reducing the risk of complications, but not for symptomatic improvement. Among the chemically defined medicines for the treatment of BPH, 5α-reductase inhibitors reduce prostate size whereas α_1_-adrenoceptor antagonists and PDE-5 inhibitors do not. By contrast, α1-adrenoceptor antagonists and PDE-5 inhibitors have a rapid and robust effect on LUTS, while the improvement of LUTS is delayed and limited with 5α-reductase inhibitors (Bschleipfer et al., 2023; Michel et al., 2023). Obviously, pharmacological mechanisms unrelated to prostate size are most relevant for reduction of symptoms.

One of the suggested mechanisms of action of *Sabal serrulata* fruit extracts is inhibition of 5α-reductase (Koch, 2001; Buck, 2004; Pigat et al., 2019; Csikós et al., 2021). Multiple studies have reported a concentration-dependent and non-competitive inhibition of 5α-reductase activity by hexane, ethanol and supercritical CO_2_ *Sabal serrulata* extracts (European Medicines Agency, 2015). Of note, the potency of *Sabal serrulata* fruit extracts to inhibit 5α-reductase differs markedly between them (Scaglione et al., 2008). However, the more relevant question is whether and to which extent such inhibition of 5α-reductase activity contributes to their clinical effects. Our data argue against a relevant contribution of this mechanism of action in the sulpiride BPH model because the effects of the *Sabal serrulata* extracts tested here differed from those of finasteride for most outcome parameters.

As reported by others (Van Coppenolle et al., 2000; Van Coppenolle et al., 2001), treatment with sulpiride for 30 days approximately doubled the weight of the lateral prostate lobes while not affecting that of the ventral and dorsal lobe or total prostate/body weight ratio.

In contrast to finasteride, WS^®^ 1541 did not inhibit the sulpiride-induced enlargement of the lateral prostate lobe (Figure 1). On the other hand, WS^®^ 1541 at doses of 600 and 900 mg/kg/day has previously been reported to reduce prostate size in the mouse model of transgenic overexpression of prolactin (Pigat et al., 2019). While these data appear contradictory, this may be related to anatomical differences between mouse and rat prostate. Recently, Dos Santos et al. published that cell plasticity in the above-mentioned mouse model drives amplification of an androgen-independent epithelial cell population which is sensitive to antioxidant therapy indicating different states of disease progression (Dos Santos et al., 2023). This indicates different stages of disease development and progression which might be targeted by different treatment options or the combination of different treatments. This may be another explanation why prostate size, particularly the enlargement of the lateral lobes, was not affected in our sulpiride study in contrast to the study published by Pigat et al. (Pigat et al., 2019). More importantly, *Sabal serrulata* fruit extracts did not reduce prostate volume or prostate specific antigen levels in serum in clinical studies in BPH patients (Carraro et al., 1996), which sharply contrast the consistently observed reduction of prostate size and prostate specific antigen upon treatment with finasteride (Rittmaster, 1994).

The lack of effect on prostate size notwithstanding, WS^®^ 1541 had several growth-related effects *in vivo* and *in vitro*. Thus, WS^®^ 1541 reduced the mRNA expression of several growth factors in the sulpiride model, including *Vegfa*, *Egf*, *Egr1*, *Icam1, Tgfb* and *Fgf2* (Figure 2), and diminished epithelial thickness in lateral lobes of sulpiride treated rats as does finasteride. This is in line with the data from the transgenic mouse model (Pigat et al., 2019). However, finasteride had minor effects on these factors but tended to decrease *Egf1* expression and to increase *Tgfb* and *Egr1* expression. On a cellular level, the extract combination WS^®^ 1541 promoted apoptosis of BPH-1 cells *in vitro*. This activity was apparently contributed by the Urtica extract WS^®^ 1031 whereas the Sabal extract WS^®^ 1473 had no effect on apoptosis (Figures 4C, D). Interestingly, in our experimental setting, we saw no induction of apoptosis by finasteride. Cell cycle assessment revealed that finasteride prevented cell cycle progression by holding the cells in G_1_-phase. A similar effect was observed by treating BPH-1 cells with WS^®^ 1541, possibly reflecting synergistic effects of WS^®^ 1031 and WS^®^ 1473 (Figures 4E, F).

### 4.3 Inflammation and oxidative stress

Inflammation and oxidative stress are tightly intertwined and are considered to be important pathophysiological factors in human BPH (Ficarra et al., 2014; Minciullo et al., 2015; De Nunzio et al., 2020). The aging prostate stroma is characterized by upregulation of several chemokines and cytokines as IL-1β, IL-2, IL-6 IL-8, IL-15 and IL-17 (Chughtai et al., 2011; De Nunzio et al., 2016). Tissue microarrays from prostatic specimen obtained from 282 BPH patients showed that the majority had infiltration of both T and B lymphocytes as well as macrophages (Robert et al., 2009). Inflammatory mediators secreted by prostatic stroma can promote slow but constant proliferation of both epithelial and stromal fibroblasts. More importantly, the International Prostate Symptom Score (IPSS) and prostate volume were higher in patients with high-grade prostatic inflammation (Robert et al., 2009).

Accordingly, several previous studies have reported anti-inflammatory effects of *Sabal serrulata* fruit extracts (Latil et al., 2015) and specifically of the combined WS^®^ 1541 extract (Pigat et al., 2019) and its WS^®^ 1473 component (Saponaro et al., 2020). Our present data confirm and extend those observations in several ways. WS^®^ 1541 reduced the mRNA expression of various inflammatory mediators including *Il1b*, *Il2*, *Il4*, *Il6*, *Kc*, *Il15*, *TNFa* and *Ikbkb* as well as *Cox2* and *Nos2* in the sulpiride model (Figure 2). Thus, WS^®^ 1541 exhibited an anti-inflammatory effect. Interestingly, finasteride increased expression of some inflammatory marker, especially *Il4*, *Il6*, *Il18*, *Ccl2* and *Ccl7* (Figure 2). This is consistent with an increased prevalence of intraprostatic inflammation especially in benign areas found in cancer patients treated with finasteride (Murtola et al., 2016).

Inflammation processes during BPH pathogenesis can cause the generation of free radicals and an impaired efficiency of antioxidant defense mechanisms. The balance between ROS and the antioxidant component also has a role in developing prostate disease (Minciullo et al., 2015). WS^®^ 1541 and its components WS^®^ 1031 and WS^®^ 1473 concentration-dependently reduced ROS in BPH-1 cells (Figure 4). Accordingly, we found diminished abundance of oxidized proteins in sections of WS^®^ 1541 treated BPH rats showing a relevant antioxidative potential of WS^®^ 1541 *in vivo* (Figure 3). An earlier study using BPH-1 cells reported that WS^®^ 1473 if anything increased ROS production whereas WS^®^ 1541 decreased it in the presence and absence of H_2_O_2_; however, these effects were not seen at all concentrations (1, 10 and 20 μg/mL) or all time points (3 and 24 h) (Saponaro et al., 2020).

### 4.4 Effect on epithelial-mesenchymal transition

On a cellular level, stromal cell proliferation and pro-fibrotic tissue remodeling besides epithelial proliferation result in prostate hyperplasia accompanied by LUTS. During BPH pathogenesis, the process of EMT might be a source of pathophysiological relevant stromal cells (Rodriguez-Nieves and Macoska, 2013; De Nunzio et al., 2016). In sulpiride treated rats *Tgfb* mRNA expression is induced compared to healthy animals and could be diminished by simultaneous treatment with WS^®^ 1541 but not with finasteride. Members of the TGF-β family are key players in EMT (Katsuno and Derynck, 2021). During EMT, epithelial cells adapt the pro-migratory phenotype of mesenchymal cells, which we mimicked in the gap-closing assay using BPH patient-derived luminal epithelial cell line BPH-1. We did not see any effect on phalloidin staining in treated BPH-1 cells ruling out possible inhibitory effects of the extracts on f-actin polymerization that could have affected migratory potential of BPH-1 cells (Supplementary Figure S5). WS^®^ 1031 was able to reduce migration activity of BPH-1 cells assumingly suppressing EMT. As WS^®^ 1473 had no effect in this assay, the activity to inhibit cell migration seen for the combination of these extracts in WS^®^ 1541 appears to be contributed by WS^®^ 1031 (Figure 5). We investigated the mRNA expression of the TGF-β isoform 1, 2 and 3 and TGF-β receptor 1 and 2 in dependence of WS^®^ 1031 and WS^®^ 1473 treatment, showing a moderate decrease in expression of *TGFB1*, *TGFB2*, *TGFB3* and *TGFBR1* upon WS^®^ 1031 treatment but without a clear concentration-response relationship. WS^®^ 1473 did not affect *TGFB1*, *TGFB3* or *TGFBR1* but concentration-dependently reduced *TGFB2* expression. Both WS^®^ 1031 and WS^®^ 1473 had a minor effect on the expression of *TGFBR2* (Figures 6B–E). Taking into account that the TGF-β1 isoform is majorly expressed in BPH-1 cells (Figure 6A), inhibitory effects on migration might be accounted for by its attenuated expression due to WS^®^ 1031. Interestingly, ROS are able to activate TGF-β from latent TGF-β binding proteins triggering downstream signaling and thus expression of ECM proteins and EMT supporting genes (Morry et al., 2017; Katsuno and Derynck, 2021).

### 4.5 Conclusions

Our combined *in vivo* and *in vitro* data demonstrate growth inhibiting effects of WS^®^ 1541 on prostate epithelial cells albeit this did not translate into a diminished prostate lobe size in the rat sulpiride model. Moreover, we found anti-inflammatory effects and reduction of markers of oxidative stress. Importantly, our *in vitro* experiments demonstrated that each extract has a specific effect profile. Thus, *Sabal serrulata* extract WS^®^ 1031 reduced ROS and attenuated cell migration without affecting f-actin polymerization. On the other hand, *Urtica dioica* extract WS^®^ 1473 also reduced ROS but in addition stimulated apoptosis. The combination of WS^®^ 1031 and WS^®^ 1473 in WS^®^ 1541 synergistically hindered cell cycle progression by impeding the entry in the S-phase. This data supports a scientific rationale of combining WS^®^ 1473 and WS^®^ 1031 in the clinically used WS^®^ 1541.

## Data Availability

The original contributions presented in the study are included in the article/supplementary material, further inquiries can be directed to the corresponding author.
