# Supplementary material for: Effects of a proprietary mixture of extracts from Sabal serrulata fruits and Urtica dioica roots (WS® 1541) on prostate hyperplasia and inflammation in rats and human cells
Source: Front Pharmacol. 2024 Mar 15;15:1379456. doi: 10.3389/fphar.2024.1379456 (PMC10979176; doi:10.3389/fphar.2024.1379456)
Supplement: Supplementary file 1 [file DataSheet1.pdf]

Supplementary Material & Methods

Materials & Methods

The extracts WS® 1473 (*Sabal serrulata*) and WS® 1031 (*Urtica dioica*) were characterized by three chemical fingerprinting methods (Supplemental figure 1), with high performance thin layer chromatography (HPTLC) and nuclear magnetic resonance spectroscopy (NMR) used for both extracts, while gas chromatography flame ionization detector (GC-FID) was exclusively used for Sabal extract and liquid chromatography high resolution mass spectrometry (LC-HRMS) exclusively used for Urtica extract due to the respective phytochemical nature of the two extracts. The aforementioned characterization of the plant material, extraction process and final extract comply in most instances to the requirements of the Consensus statement on the Phytochemical Characterization of Medicinal Plant extracts (Heinrich et al 2022).

HPTLC

HPTLC analysis was carried out using HPTLC 60 F254 (Merck) plates. The samples were dissolved at a concentration of ca. 100 mg in 10 ml methanol by sonication for ca. 10 min, and subsequently 5 and 10 µL of Sabal and Urtica solution, respectively, was spotted on the plates by a CAMAG Automatic TLC Sampler 4. Prior to analysis, a CAMAG Automatic Developing Chamber 2 was conditioned with the elution solvent for 10 min and the humidity was subsequently equilibrated with a saturated $MgCl_2 \cdot 6\ H_2O$ solution for another 10 min. The HPTLC plates were conditioned in the Chamber for 2 min prior to analysis. For the Sabal analysis, the elution solvent was composed as follows: heptane/ethyl acetate/acetic acid = 70/30/1 (v/v/v). For the Urtica analysis, the elution solvent was composed as follows: toluene/ethyl acetate/acetic acid/methanol = 56/30/10/4 (v/v/v/v). The separation runs took approximately 30 min to reach 6 cm of separation length. After the run, the elution solvent was actively evaporated by an air stream for 5 min at room temperature, and the plates were sprayed with a staining reagent which was prepared as follows: 20 ml deionized water, 35 ml 85 % (m/m) phosphoric acid, 1 g vanillin (Sigma Aldrich), 2.5 ml 96 % (v/v) ethanol were mixed until vanillin was completely dissolved, and subsequently the solution was diluted with deionized water ad 100 ml. The staining was developed by placing the plates for ca. 2–3 min on a hot plate tempered to 125°C. Visualization at visible light and under UV radiation at 366 nm wavelength was performed with a CAMAG TLC Visualizer 2 using CAMAG visionCATS v2.5 software.

NMR spectroscopy

NMR spectra were acquired with a 600 MHz Bruker Avance III HD spectrometer equipped with an inverse TCI Prodigy cryo probe. Prior to measurement, 30.2 mg of Sabal extract and 30.0 mg of Urtica extract were dissolved each in 600 µL $DCCl_3$ and DMSO-d_6, respectively. Tetramethylsilane was used as internal standard for both samples. 1H spectra were recorded with 32 accumulated scans with a spectral width of 18 ppm, a transmitter frequency offset of 8 ppm, and a digital resolution of 64 k data points. 13C spectra were recorded with 12 k accumulated scans with a spectral width of

240 ppm, a transmitter frequency offset of 110 ppm, and a digital resolution of 64 k data points. Both samples were tempered to 25°C during measurement. The spectra were processed with an exponential window function with a line broadening of 0.3 Hz and 1.0 Hz for ^{1}H and ^{13}C, respectively. The spectra were recorded and processed using Bruker Topspin v3.5pl7 software and analyzed and visualized using ACD/Labs Spectrus Processor v2021.2.0.

GC-FID

The GC-FID analysis was performed according to the protocol as described in the European Pharmacopoea (Ph. Eur. 11.0). The chromatograms were recorded and processed using Thermo Chromeleon v7.2.10ES software and analyzed and visualized using ACD/Labs Spectrus Processor v2021.2.0.

LC-HRMS

The LC-HRMS analysis was carried out on a Thermo Vanquish UHPLC coupled to a Thermo Orbitrap Fusion mass detector by an electrospray source (ESI). Urtica extract was dissolved at a concentration of 1 mg/ml in 50 % (v/v) methanol and 20 µL of the sample solution were injected. A Waters Acquity UPLC HSS T3 1.8 µm, 2.1x150 mm column was used for separation. The eluents were composed as follows: Eluent A: 97.4 % H2O, 2.5 % acetonitrile, 0.1 % formic acid; Eluent B: 97.4 % acetonitrile, 2.5 % H2O, 0.1 % formic acid. The elution gradient was as follows: 0–2 min 0 % B, 2–25 min. linear from 0–80 % B, 25–26 min. linear from 80–100 % B, 26–31 min. 100 % B, 31–32 min. linear from 100 – 0 % B, 32–35 min. 0 % B. The positive and negative full scan chromatograms were acquired with an orbitrap mass resolution of 120 000. The chromatograms were recorded and processed using Thermo Xcalibur v4.5.474.0 software and analyzed and visualized using ACD/Labs Spectrus Processor v2021.2.0.

Supplementary Figures

Supplementary Figure 1

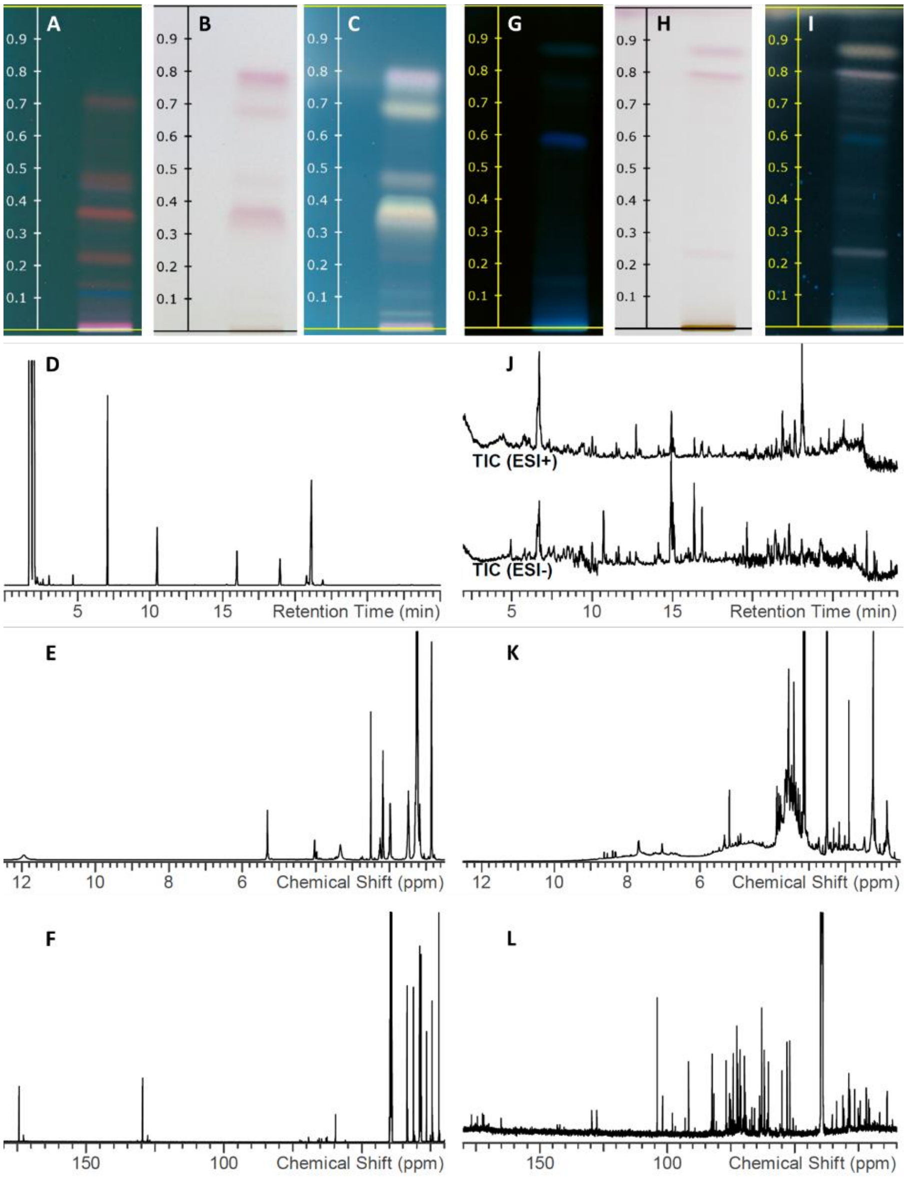

Supplementary Figure 1: Analytical Fingerprints of the two individual components of Prostagutt, WS® 1473 and WS® 1031. (A) HPTLC fingerprint of WS® 1473 at 366 nm. (B) HPTLC fingerprint like A, derivatized with Vanillin/H_3PO_4 at visible light. (C) HPTLC fingerprint like B, at 366 nm. (D) GC-FID fingerprint of WS® 1473 acquired according to Ph. Eur. 11.0. (E) ^{1}H-NMR fingerprint of WS® 1473 acquired in $DCCl_3$. (F) ^{13}C-NMR fingerprint of WS® 1473 acquired in $DCCl_3$. (G) HPTLC fingerprint of WS® 1031 at 366 nm. (H) HPTLC fingerprint like G, derivatized with Vanillin/H_3PO_4 at visible light. (I) HPTLC fingerprint like H, at 366 nm. (J) ESI-LC-HRMS total ion current chromatogram fingerprints of WS® 1031 in positive (TIC (ESI+)) and negative (TIC (ESI-)) ion mode. (K) ^{1}H-NMR fingerprint of WS® 1031 acquired in DMSO-d6. (L) ^{13}C-NMR fingerprint of WS® 1031 acquired in DMSO-d6.

Supplementary Figure 2

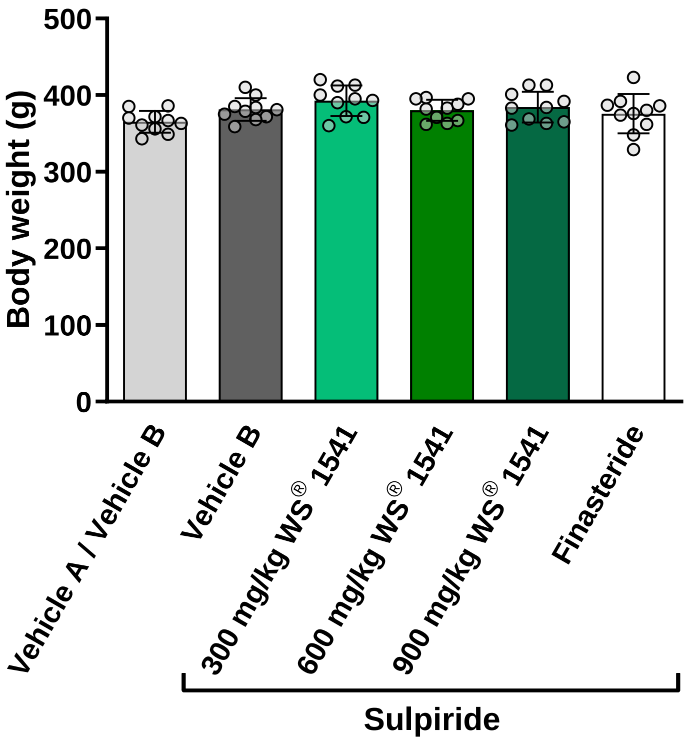

Supplementary Figure 2. (A) Body weights of rats after 30 days of daily i.p. administration with Sulpiride (40 mg/kg) or vehicle A (acetic acid 1 %) and oral treatment with vehicle B (0.2 % agar suspension with 1 % Tween 80 in distilled water), different dosages of WS® 1541 (300, 600 and 900 mg/kg) or finasteride. Each circle represents one rat (n = 10), and bars represent mean ± SD.

Supplementary Figure 3

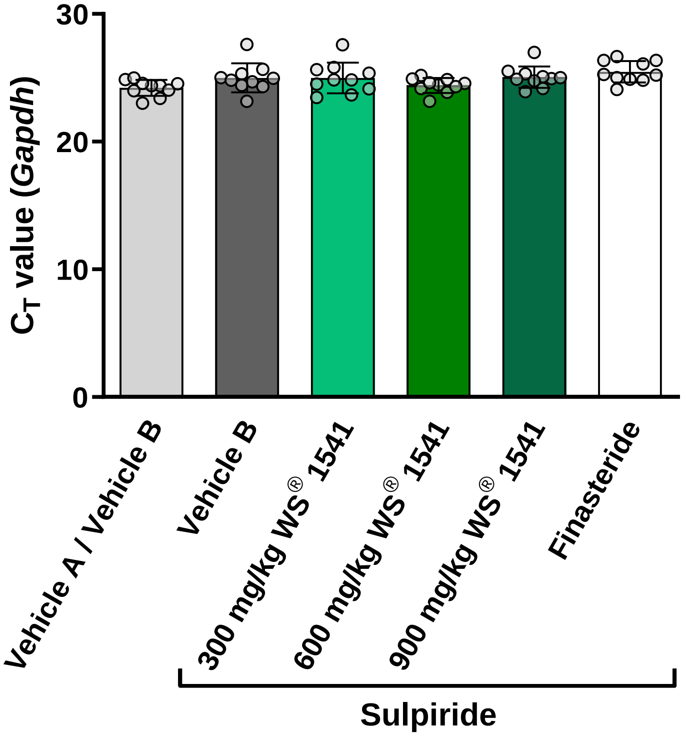

Supplementary Figure 3. Assessment of C_T values of *Gapdh* shows similar expression levels of this reference gene among all various treatment groups. Each circle represents one rat (n = 10), and bars represent mean ± SD.

Supplementary Figure 4

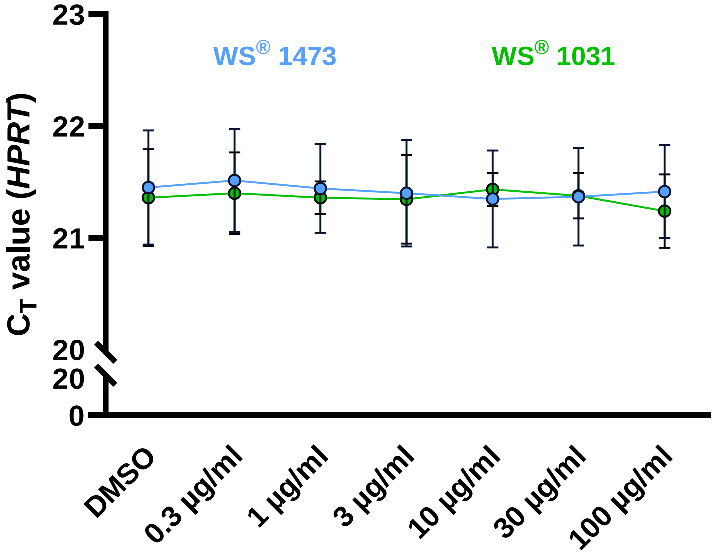

Supplementary Figure 4. Assessment of C_T values of *HPRT* showing constant expression levels in all different treatment groups. Data is expressed as mean ± SD, n = 5 independent experiments.

Supplementary Figure 5

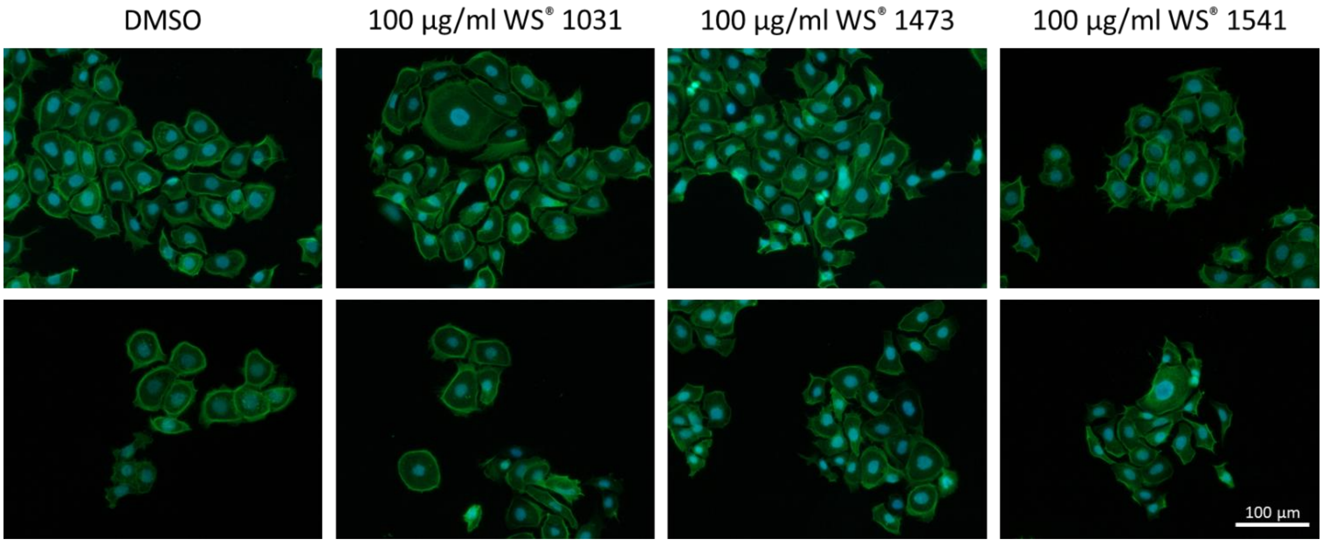

Supplementary Figure 5. Phalloidin-Atto 488 stained F-actin in BPH-1 cells treated with DMSO as control or with 100 µg/ml WS® 1031 and WS® 1473 or WS® 1541. Phalloidin appears in green, nuclei are stained in blue with DAPI (Scale: 100 µm).
